# Corrigendum to “Developing and using hints in computerized dynamic assessment of a TOEFL *iBT* reading exam” <[Heliyon 6 (9) (2020) e04985]>

**DOI:** 10.1016/j.heliyon.2022.e11453

**Published:** 2022-11-11

**Authors:** Sahbi Hidri, Leila Fekri Pileh Roud

**Affiliations:** a Department of Education, Abu Dhabi Women's Campus, Higher Colleges of Technology, Abu Dhabi, United Arab Emirates; b University of Tehran, Kish International Campus, Iran

In the original published version of this article, Dr. Sahbi Hidri's affiliation was not correct, the author confirms that this paper was published while employed at the Higher Colleges of Technology, Abu Dhabi. The affiliation of Dr. Sahbi Hidri has been changed from “Department of English- Faculty of Human & Social Sciences of Tunis- University of Tunis, Tunis, Tunisia” to “Department of Education, Abu Dhabi Women's Campus, Higher Colleges of Technology, Abu Dhabi, UAE”. The authors apologize for the errors. Both the HTML and PDF versions of the article have been updated to correct the errors.

